# *H. pylori* attenuates TNBS-induced colitis via increasing mucosal Th2 cells in mice

**DOI:** 10.18632/oncotarget.17962

**Published:** 2017-05-18

**Authors:** Yi-Zhong Wu, Gao Tan, Fang Wu, Fa-Chao Zhi

**Affiliations:** ^1^ Guangdong Provincial Key Laboratory of Gastroenterology, Department of Gastroenterology, Nanfang Hospital, Southern Medical University, Guangzhou, China; ^2^ Department of Gastroenterology, Hunan Provincial People’s Hospital, Changsha, China; ^3^ Department of Gastroenterology, The First Affiliated Hospital of Wenzhou Medical University, Wenzhou, China

**Keywords:** H. pylori, Crohn’s disease, mucosal immunology, Th cells

## Abstract

There is an epidemiological inverse relationship between *Helicobacter pylori* (*H. pylori*) infection and Crohn’s disease (CD). However, whether *H. pylori* plays a protective role against CD remains unclear. Since 2, 4, 6-trinitrobenzene sulfonic acid (TNBS)-induced colitis is thought to resemble CD, we investigated whether *H. pylori* can attenuate TNBS-induced colitis in mice. Here we show that *H. pylori* can attenuate the severity of TNBS-induced colitis. In addition, *H. pylori* not only down-regulates Th17 and Th1 cytokine expression, but can up-regulate Th2 cytokine expression and increase the Th2:Th17 ratio of CD4^+^ T in the colonic mucosa of TNBS-induced colitis. Our results indicate that *H. pylori* attenuates TNBS-induced colitis mainly through increasing Th2 cells in murine colonic mucosa. Our finding offers a novel view on the role of *H. pylori* in regulating gastrointestinal immunity, and may open a new avenue for development of therapeutic strategies in CD by making use of asymptomatic *H. pylori* colonization.

## INTRODUCTION

Crohn’s disease (CD), one type of inflammatory bowel disease (IBD) [1], is a life-long, chronic and relapsing disease that may occur anywhere in the gastrointestinal tract [2, 3]. Although its pathogenesis is still poorly understood, genetic predisposition and environment, most notably the gut microorganism were thought to be the important aetiology [4]. However, up to now there is no decisive evidence of an etiologic role for any specific microbe. Whether there is a still unidentified specific microbe implicated in the pathogenesis of CD deserves to be explored.

*Helicobacter pylori* (*H. pylori*), a highly adaptive gram-negative bacterium colonized the human gastric mucosa, has co-existed with humans for over 50,000 years [5, 6]. Although it is positively correlated with the pathogenesis of gastric ulcers, gastric cancers and mucosa-associated lymphoid tissue lymphoma, less than 15% and 1% of infected patients will develop gastric ulcer and cancer, respectively [7, 8]. Interestingly, *H. pylori* was recently isolated and detected in the intestinal mucosa of patients with CD [9]. Additionally, many recent studies have reported that CD patients have lower prevalence of *H. pylori* infection [10–12]. Furthermore, some reports have shown that there are rapid and clinical onset of CD after eradication of *H. pylori* infection [13, 14]. Although these researches found an inverse relationship between *H. pylori* infection and CD, whether *H. pylori* plays a protective role against CD and the potential protective mechanism remain unclear.

The purpose of this study was to determine whether *H. pylori* plays a protective role against CD and the potential protective mechanism. For this purpose, we used a murine model of CD-like colitis induced by 2, 4, 6-trinitrobenzene sulfonic acid (TNBS) as described previously [15].

## RESULTS

### Administration of *H. pylori* attenuates the severity of TNBS-induced colitis

**Figure 1:**
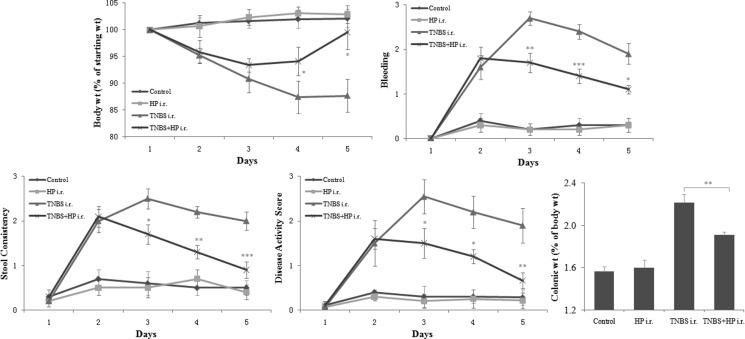
Mice receiving intrarectal (i.r.) administration of NCTC11639 displayed less weight loss, less bleeding, greater stool consistency and low disease activity scores and had decreased colonic:body weight Mice were treated with 2.5%TNBS (day 1) with and without NCTC11639 (days 2–4) via an i.r. route in TNBS+ NCTC11639 and TNBS groups, respectively. Mice were treated with saline as control. Data are shown as the mean ± SD from 10 mice per group. *P < 0.05, **P < 0.01, ***P < 0.001 vs TNBS groups.

In order to determine whether *H. pylori* attenuates the severity of TNBS-induced colitis, we treated mice with NCTC11639 (one *H. pylori* strain) after administration of TNBS enema and compared the disease activity scores, the colonic weight changes and the macroscopic and microscopic appearances of colons between mice treated with TNBS plus NCTC11639 and mice treated with TNBS only. We found that after administration of NCTC11639 enema, the TNBS-treated mice displayed less weight loss, less bleeding, greater stool consistency and low disease activity scores and had decreased colonic:body weight (Figure 1).

**Figure 2:**
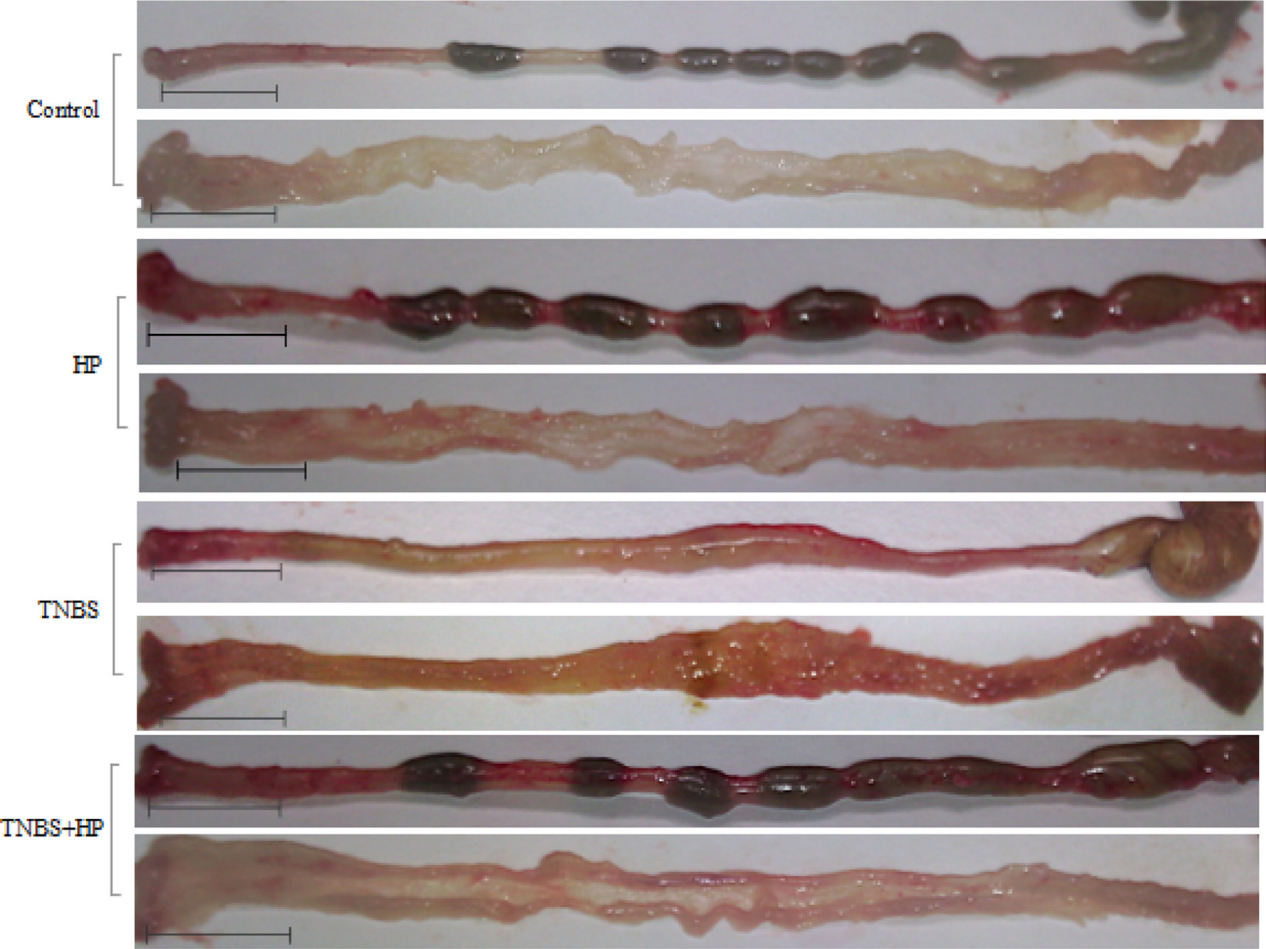
Macroscopic appearance of colons from Control (saline-treated mice), HP-treated mice, TNBS-treated mice and TNBS + NCTC11639-treated mice TNBS-treated colons display thickened edematous walls and mucosal ulcerations, especially at distal 4–6 cm from the anus, while these changes are ameliorated in TNBS + NCTC11639-treated mice. Bars represent 1 cm.

**Figure 3:**
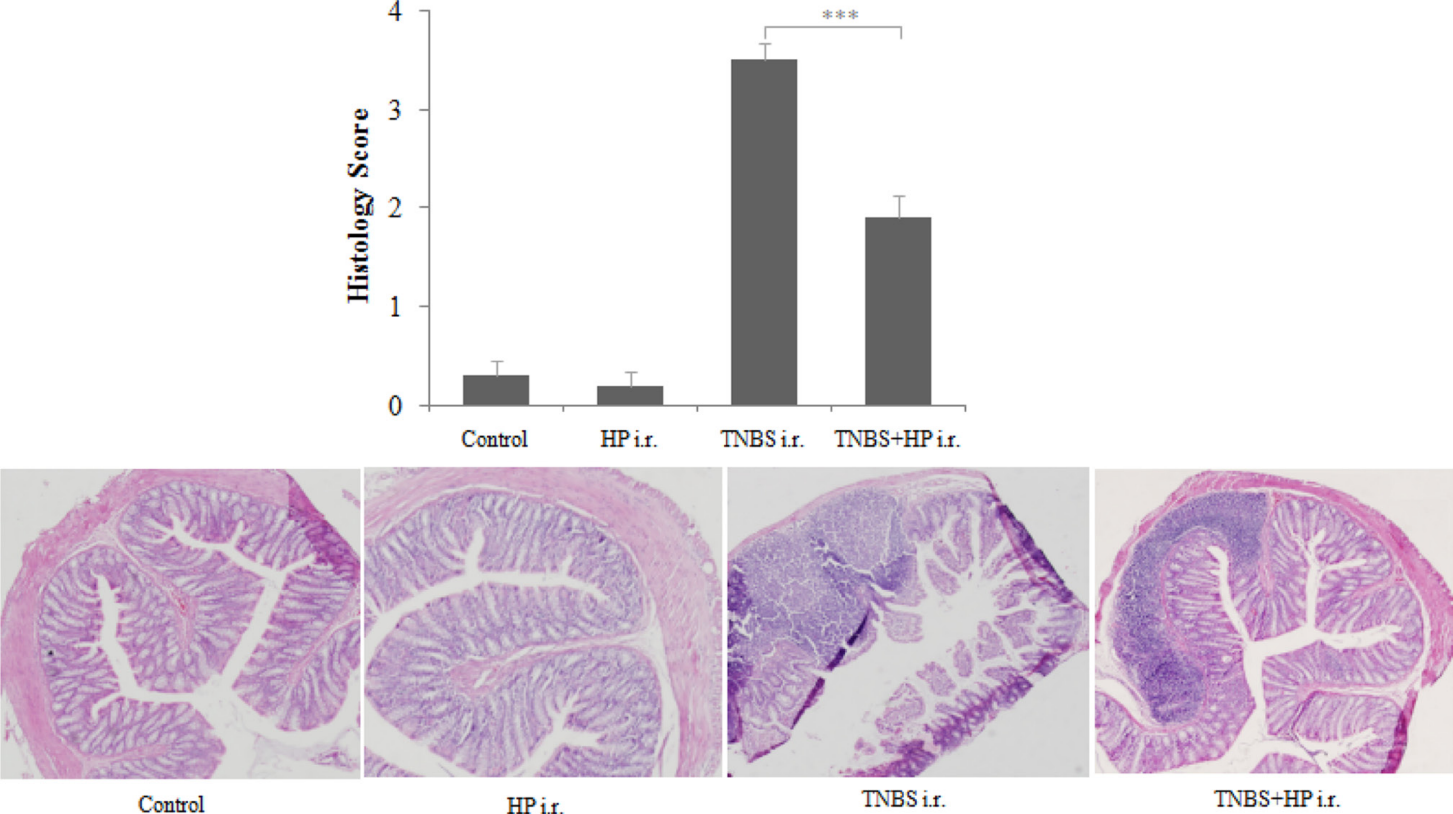
Microscopic appearance of colons from Control, HP-treated mice, TNBS-treated mice and TNBS + NCTC11639-treated mice Paraffin-embedded sections of the distal 4–6 cm from the anus were stained with HE. A, no significant inflammation in the bowel wall. B, severe transmural inflammation in the bowel wall. C, moderate inflammation in the bowel wall. All sections are shown at 100× magnification.

Consistent with the weight changes in the *H. pylori*-treated mice, we found that the TNBS-treated mice displayed less thickened edematous colonic walls and less mucosal ulcerations after administration of NCTC11639 enema, especially at distal 4–6 cm from the anus (Figure 2). In line with the macroscopic appearances of colons, we found that the mice treated with TNBS+NCTC11639 had less histological scores and displayed alleviated degree of inflammation of the colonic walls compared with the mice treated with TNBS only (Figure 3). Together, these results confirm that administration of *H. pylori* can attenuate the severity of TNBS-induced colitis.

### Administration of *H. pylori* down-regulates Th17 and Th1 cytokine expression, but up-regulates Th2 cytokine expression in murine colonic mucosa

Since abnormal T helper (Th) lymphocyte responses have been considered to play critical roles in the pathogenesis of IBD, we subsequently investigated whether *H. pylori* attenuates the severity of TNBS-induced colitis through regulating the Th lymphocyte responses. To test this, we treated mice with NCTC11639 after administration of TNBS enema and compared the colonic mRNA expression of multiple inflammation-mediating cytokines involved in Th17, Th1 and Th2 immune responses between mice treated with TNBS plus NCTC11639 and mice treated with TNBS only. We found that the colonic mRNA expression of Th17-related cytokines (IL-23p19, IL-17A, IL-6, IL-1β, TGF-β and STAT-3) was significantly lower in the mice treated with TNBS+NCTC11639 than in the mice treated with TNBS only (Table 1). Similarly, the colonic mRNA expression of Th1-related cytokines (IL-12p35, TNF-α, IFN-γ and STAT-1) was significantly lower in the mice treated with TNBS+NCTC11639 than in the mice treated with TNBS only (Table 1). However, the changes of Th1-related cytokines were far weaker than the changes of Th17-related cytokines.

**Table 1:** Th-related gene expression in mouse colonic mucosa detected by real-time PCR

Gene	Control	NCTC11639	TNBS	TNBS+NCTC11639
IL-12p35	1 ± 0.06	0.90 ± 0.06	1.35 ± 0.06	0.63 ± 0.08^*^
TNF-α	1 ± 0.06	0.93 ± 0.14	2.76 ± 0.44	1.23 ± 0.10^*^
IFN-γ	1 ± 0.08	0.91 ± 0.08	1.59 ± 0.01	0.32 ± 0.01^**^
STAT-1	1 ± 0.01	0.97 ± 0.01	2.26 ± 0.04	1.44 ± 0.02^**^
IL-23p19	1 ± 0.34	1.10 ± 0.26	2.16 ± 0.01	1.81 ± 0.01^**^
IL-17A	1 ± 0.15	1.22 ± 0.24	5.15 ± 0.22	1.11 ± 0.03^*^
IL-6	1 ± 0.25	0.89 ± 0.35	5.79 ± 0.54	0.61 ± 0.02^*^
IL-1β	1 ± 0.52	0.85 ± 0.14	30.27 ± 10.66	0.86 ± 0.19^*^
TGF-β	1 ± 0.06	1.21 ± 0.09	1.82 ± 0.15	1.21 ± 0.15^*^
STAT-3	1 ± 0.03	0.93 ± 0.05	3.47 ± 0.12	0.67 ± 0.08^**^
IL-4	1 ± 0.02	1.96 ± 0.03	0.25 ± 0.03	4.71 ± 0.20^*^
IL-5	1 ± 0.03	1.57 ± 0.04	0.27 ± 0.04	0.85 ± 0.01^*^
IL-10	1 ± 0.20	1.17 ± 0.20	0.32 ± 0.03	0.82 ± 0.01^**^
STAT-6	1 ± 0.08	1.21 ± 0.08	0.18 ± 0.01	0.26 ± 0.02^*^

Relative mRNA levels are normalized to β-actin and expressed as a fold relative to the Control groups. Data are shown as the mean ± SD from 10 mice per group. *P < 0.05,**P < 0.01 vs TNBS groups.

Interestingly, we found that the colonic mRNA expression of Th2-related cytokines (IL-4, IL-5, IL-10 and STAT-6) was significantly higher in the mice treated with TNBS+NCTC11639 than in the mice treated with TNBS only (Table 1). Together, these results provide evidence that *H. pylori* can attenuate the severity of TNBS-induced colitis mainly through regulating the Th17 and Th2 lymphocyte responses.

### Administration of *H. pylori* increases the Th2:Th17 ratio of CD4^+^ T in murine colonic mucosa

**Figure 4:**
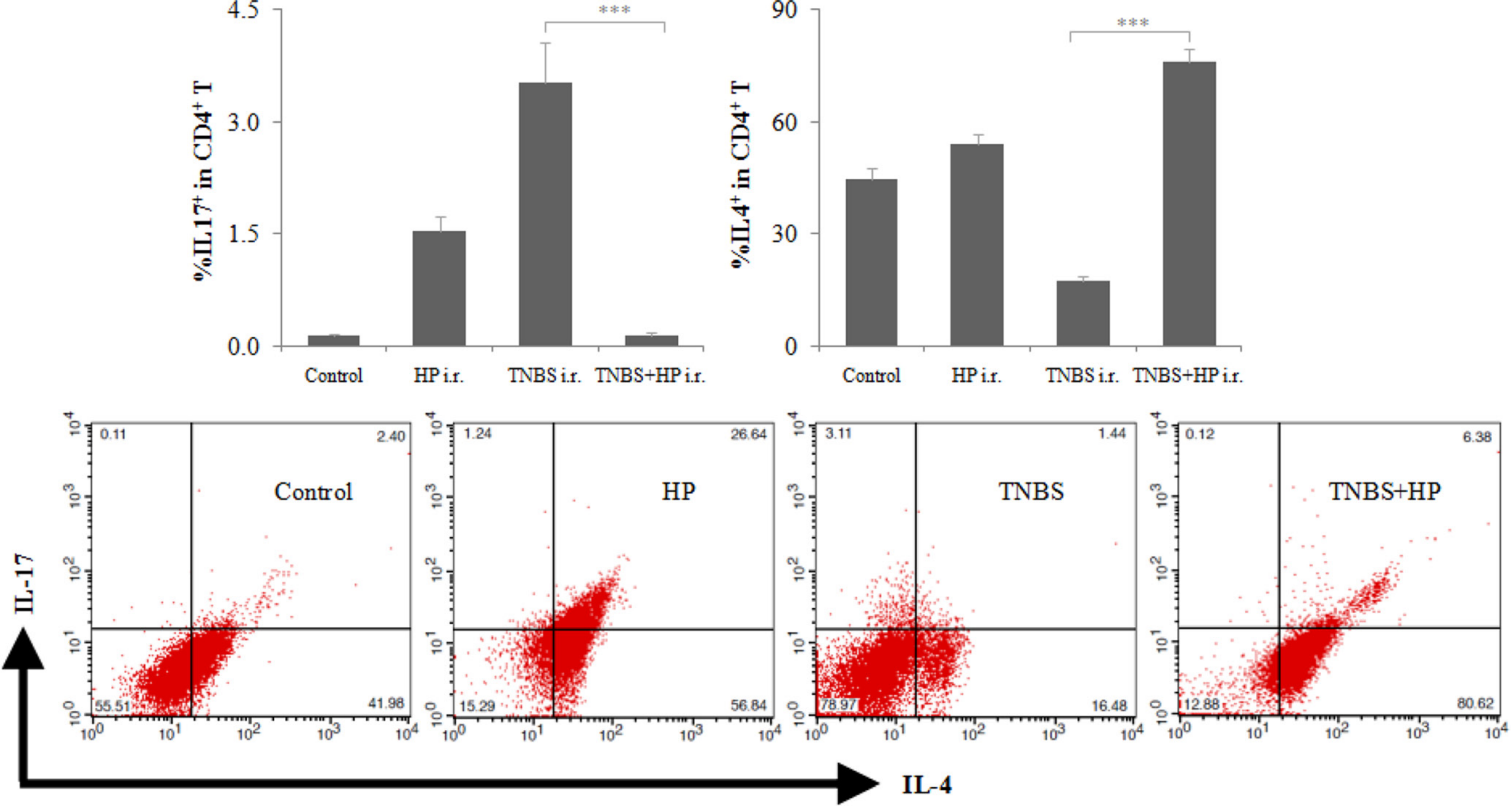
Intrarectal (i.r.) administration of NCTC11639 increases the Th2:Th17 ratio of CD4^+^ T in murine colonic mucosa Mice were first treated with saline (Control), HP, TNBS or TNBS+NCTC11639, then isolated CD4^+^ T in murine colonic mucosa and finally determined the levels of Th17 and Th2 cells by FCM. Upper, percentage of IL-17^+^ and IL-4^+^ cells in CD4^+^ T cells. Bottom, representative plots. Data are shown as the mean ± SD from 10 mice per group. ***P < 0.001 vs TNBS groups.

To further elucidate the potential mechanism by which *H. pylori* attenuates the severity of TNBS-induced colitis, we treated mice with NCTC11639 after administration of TNBS enema, then isolated CD4^+^ T in murine colonic mucosa and finally determined the levels of Th17 and Th2 cells by FCM. We found that the mice treated with TNBS+NCTC11639 had decreased levels of Th17 cells, increased levels of Th2 cells and increased Th2:Th17 ratio of CD4^+^ T in colonic mucosa compared with the mice treated with TNBS only (Figure 4). These results suggest that *H. pylori* might inhibit and promote the differentiation of Th17 and Th2 to attenuate the severity of TNBS-induced colitis, respectively.

## DISCUSSION

This study is focused on exploring whether *H. pylori* plays a protective role against CD and the potential protective mechanism. Our results in a murine model of TNBS-induced CD-like colitis show that *H. pylori* enema plays a protective role against CD. We found that *H. pylori* enema can attenuate the severity of TNBS-induced colitis from the macroscopic and microscopic appearances of murine colons.

In further experiments concerning the protective mechanism by which *H. pylori* plays a protective role against CD, our data show that *H. pylori* enema can regulate the Th17, Th1 and Th2 lymphocyte responses to attenuate the severity of TNBS-induced colitis. We found that *H. pylori* not only down-regulated the Th17 cytokine expression and to a lesser extent, the Th1 cytokine expression, but also up-regulated Th2 cytokine expression in murine colonic mucosa. Moreover, *H. pylori* can increase the Th2:Th17 ratio of CD4^+^ T in murine colonic mucosa. These findings suggest that *H. pylori* might inhibit and promote the differentiation of Th17 and Th2 to attenuate the severity of TNBS-induced colitis, respectively.

Recent studies found that abnormal Th17 responses have vital roles in the pathogenesis of many chronic inflammatory diseases, including CD [16–19]. As the advances in understanding the function of Th17 cells, the Th1/Th2 paradigm has now been replaced by the new Th1/Th2/Th17 paradigm. Here we found that *H. pylori* can down-regulate Th17 profile and Th1 profile, but up-regulate Th2 profile. In addition, some studies found that the Th2 profile may be important in balancing the immune response [14, 20]. Therefore, it is feasible that *H. pylori* offers protective roles against CD by increasing Th2 profile to balance Th1/Th2/Th17 cell responses.

In summary, this is the first study to demonstrate that *H. pylori* attenuates TNBS-induced colitis mainly through increasing the Th2 cells in murine colonic mucosa. This finding is significant because it not only can offers a novel view on the role of *H. pylori* in regulating gastrointestinal immunity, but also may open a new avenue for development of therapeutic strategies in CD by making use of asymptomatic *H. pylori* colonization.

## MATERIALS AND METHODS

### Animals

Male BALB/c mice aged 6–8 weeks were purchased from the Department of Laboratory Animal Center of Southern Medical University, housed and fed in the animal maintenance facility at Southern Medical University. All animal experiments were reviewed and approved by the Experimental Animal Care and Ethical Committee at Nanfang Hospital of Southern Medical University.

### Bacterial enema preparation

*H pylori* (NCTC11639 strain) obtained from Guangdong Provincial Key Laboratory of Gastroenterology of Nanfang Hospital were inoculated on Campylobacter-selective agar (BD Biosciences) supplemented with 5% sterile horse blood, 5 μg/ml rimethoprim, 10 μg/ml vancomycin and 10 μg/ml nystatin, and cultured at 37°C in a humidified microaerophilic atmosphere with 5% O_2_, 10% CO_2_ and 85% N_2_. After 2 days, the bacteria was resuspended and diluted with sterile 0.9% saline solution. The bacterial concentration was estimated by using a spectrophotometer (1 OD_600_ = 1 × 10^9^ CFU/ml). The *H pylori* suspension at the concentration of 1 × 10^9^ CFU/ml was used as the bacterial enema.

### Animal studies

The experimental schedule is outlined in Supplementary Figure 1. For model 1, TNBS-induced colitis studies were performed as per the protocol described previously [15]. For model 2, these studies were performed as the model-1experiment, but 500 μl of *H pylori* enema was administrated into the mouse colonic lumen continuous 3 days after administration of 100 μl of TNBS solution. For models 3 and 4, these control studies only used saline and HP enemas, respectively. All harvested mice of models 1, 2, 3 and 4 were sacrificed and subsequently, their colons were intactly removed. The colons were longitudinally opened, cleaned and weighted after their length was measured. Since the distal 4–6 cm of the TNBS-treated colons were remarkably thickened and edematous, paraffin sections and total RNA isolation were taken from these regions. Paraffin sections were prepared for H&E to assess the degree of inflammation graded semi-quantitatively from 0 to 4 as previously described [21].

### Clinical assessment

Body weight (% starting weight), bleeding and stool consistency were measured as clinical parameters to calculate a disease activity score. The scoring for three parameters were as follows: % starting weight (0 = > 99%, 1 = 90–99%, 2 = 85–90%, 3 = < 85%); bleeding (0 = negative, 1 = faint blue on hemoccult, 2 = blue on hemoccult, 3 = gross red); stool consistency (0 = normal, 1 = slightly soft, 2 = loose, 3 = liquid). The average of three clinical parameters was used as the disease activity score.

### Real-time PCR

Total RNA was isolated from fresh colonic tissue using the RNAiso Plus (Takara Bio) and then cDNA synthesis was performed using the PrimeScript RT reagent Kit with gDNA Eraser (Takara Bio). Real-time PCR was performed in triplicate using the LightCycler 480 System (Roche). Each 20-µl PCR reaction contained 5 µl cDNA corresponding to 25 ng RNA as a template, 0.5 µM of each primer (Supplementary Table 1), and 1 × LightCycler 480 SYBR Green I Master (Roche). Samples were loaded into the LightCycler 480 Multiwell Plate 96 (Roche) and incubated for initial denaturation at 95°C for 10 min followed by 45 cycles, each cycle consisting of 95°C for 15 s, “touchdown” of –1°C/cycle from the start annealing temperature 65°C to the end 60°C for 5 s, and 72°C for 10 s. β-actin was used an internal standard, and ΔΔCT values were calculated to obtain fold changes relative to the control group.

### Cell isolation and flow cytometry

Lamina propria lymphocytes were isolated from fresh colonic tissue as described previously [22] and the CD4^+^ T cell population was obtained from these lymphocytes by positive selection by mouse CD4^+^ T cell isolation columns as the manufacturer’s protocol (Miltenyi Biotec). After that, these CD4^+^ T cells were incubated with 10 μg/ml BFA, 50 ng/ml PMA and 750 ng/ml Ionomycin (Sigma) at 37°C in a tissue culture incubator. Four hours later, surface and intracellular cytokine staining was performed as described previously [22]. FITC anti-mouse IL-17A and PE anti-mouse IL-4 were purchased from BioLegend. Finally, flow cytometry was performed on FACSCalibur (BD Biosciences) and analyzed using FlowJo software (Tree Star Inc.).

### Statistical analysis

The results are shown as the mean ± standard deviation. Statistical significance was determined by one-way analysis of variance with Tukey’s multiple comparisons under equal variances or with Dunnett T3’s multiple comparisons under unequal variances. a value of *P* < 0.05 was considered to be statistically significant.

## SUPPLEMENTARY MATERIALS FIGURE AND TABLE


