# Supplementary material for: H. pylori attenuates TNBS-induced colitis via increasing mucosal Th2 cells in mice
Source: Oncotarget. 2017 May 18;8(43):73810–6. doi: 10.18632/oncotarget.17962 (PMC5650302; doi:10.18632/oncotarget.17962)
Supplement: Supplementary file 1 [file oncotarget-08-73810-s001.pdf]

H. pylori attenuates TNBS-induced colitis via increasing mucosal Th2 cells in mice

Supplementary Materials

Supplementary Table 1: Real-time PCR primer sequences for selected genes

Gene	Sense	Antisense
IL-4	AAACGTCCTCACAGCAACGA	GCATCGAAAAGCCCGAAAGA
IL-5	AGGCTTCCTGTCCCTACTCA	CCCCCACGGACAGTTTGATT
IL-6	GTGGCTAAGGACCAAGACCA	ATAACGCACTAGGTTTGCCGA
IL-10	CAGTACAGCCGGGAAGACAA	CCTGGGGCATCACTTCTACC
IL-12p35	ACCCTTGCATCTGGCGTCTA	TGGTCTTCAGCAGGTTTCGG
IL-23p19	TGGAGCAACTTCACACCTCC	GGCAGCTATGGCCAAAAAGG
IL-17A	TACCTCAACCGTTCCACGTC	ATGTGGTGGTCCAGCTTTCC
IL-1β	TGCCACCTTTTGACAGTGATG	AAGGTCCACGGGAAAGACAC
TGF-β	GCTGAACCAAGGAGACGGAA	AGAAGTTGGCATGGTAGCCC
TNF-α	GGTCCCCAAAGGGATGAGAAG	TTGGTGGTTTGCTACGACG
IFN-γ	CGGCACAGTCATTGAAAGCC	TGCATCCTTTTTCGCCTTGC
STAT-1	GGATCGCTTGCCCAACTCTT	TGGTCGCAAACGAGACATCA
STAT-3	ACCAACGACCTGCAGCAATA	TCCATGTCAAACGTGAGCGA
STAT-6	GCCAGGGTTTACAGTGAAGAAG	AGCCGTTGCAGTTTTTCTGG
β-actin	CCACCATGTACCCAGGCATT	AGGGTGTAAAACGCAGCTCA

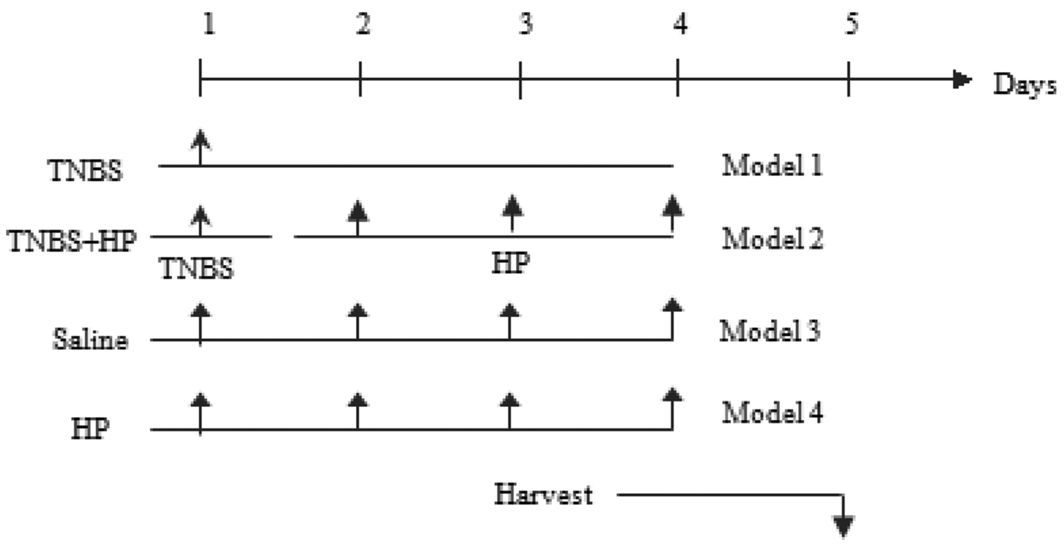

Supplementary Figure 1: Overview of models 1, 2, 3 and 4. In model 1, mice were treated with TNBS enema at day 1. In model 2, mice were treated with TNBS enema at days and HP enema at days 2, 3 and 4. In models 3 and 4, mice were treated with saline and HP enema, respectively. All mice from models 1, 2, 3 and 4 were harvested at day 5.
